# Therapeutical Action of Cold

**Published:** 1880-03

**Authors:** W. H. Thomson

**Affiliations:** Professor of Therapeutics and Materia Medica in the Medical Department of the University of the City of New York.


### ARTICLE III.

### *The Therapeutical Action of Cold.*

**BY W. H. THOMSON, M. D.**

**Professor of Therapeutics and Materia Medica in the Medical Department of the University of the City of New York.**

*Gentlemen:*—Remedial agents are of two kinds: First, drugs; and second, other therapeutic measures, such as temperature, electricity, etc. For the sake of convenience, we will here consider those remedial agents which are not drugs, and first, among them, we will study one of the physical forces or imponderables—cold.

Physically, cold is the absence of heat. Therapeutically, it is a positive agent, and has five actions:

1. Tonic.
2. Styptic.
3. Antiphlogistic.
4. Anæsthetic.
5. Antipyretic.

In the first three, cold acts only upon the vaso-motor system as pure irritant neurotic. In the last two it acts simply on physical principles.

COLD AS A TONIC.

We have said that cold, when it acts as a tonic, is an irritant Every irritant produces a shock and causes an expenditure of the energy of the part irritated. The energy of the part irritated, therefore, becomes depressed; but this depression differs from that produced by a simple sedative, in that it is followed—provided the shock is not so great as to cause exhaustion—by a *reaction* to or beyond the condition in which the part was prior to the irritation. Thus, cold, as an irritant, affects the vaso-motor system and produces a shock which is followed by a reaction. In other words, this system is exercised, and all moderate exercise tends to strengthen the organ called into action, and permanently to improve its nutrition. Cold, then, is a vascular tonic, and may be used generally or locally. When the circulation is feeble, and there is loss of muscular power, the general use of cold will arouse the heart, restore arterial tone, and thereby improve the nutrition of the whole body. For this purpose either the dip, shower, or sponge-bath may be used, according to the strength of the patient, taking care never to cause exhaustion by its too frequent or too protracted use. A thorough reaction, as indicated by a glow of the skin, should always follow the bath, and never a sensation of lassitude or fatigue. When the irritant effect produced by the cold water alone is not sufficient, salt or some mild rubefacient may be added. If the patient is too feeble to bear even the sponge-bath, simple exposure of the surface of the body to cold air will often prove beneficial. In all cases reaction may be assisted by friction with a rough towel.

A cold douche to the nape of the neck is indicated in the following conditions:

1. When, after sunsrtoke, the arteries of the head remain dilated, and there is headache and dizziness on exertion or exposure to the sun.

2. In all cases in which headache is confined to one side, and is attended by dilation of one temporal artery and suffusion of one eye.

3. In false croup, or the crowning respiration of children.

4. In tinnitus aurium, when the throbbing is synchronous with the beating of the heart, and the tympanic arteries are distended, the cold douche to the nape of the neck, aided by the internal use of hydrobromic acid, may afford relief.

Sponging the chest of a phthisical patient with cold water lessens the susceptibility to cold.

Local applications of cold water are useful in promoting absorption of inflammatory effusions and exudations in the subacute and cronic stages; also in restoring the balance of the circulation in the liver and spleen when enlarged in malarial poisoning.

The hip or sitz-bath is useful in hemorrhoids, prolapse of the rectum, and congestion of the pelvic viscera.

COLD AS A STYPTIC.

As a styptic, cold acts by constringing the arteries through its influence on the vaso-motor nerves. It is preferable to astringent drugs or other hæmostatics, because it obviates the necessity of applying irritant substances to the bleeding part. Nor need the cold always be applied directly to the seat of the hemorrhage; for it will also affect distant parts in accordance with the laws of the vaso-motor system, the most important of which are the following:

*First.*—An impression on the afferent nerves of a given part will cause a variation in the calibre of the arteries of that part.

*Second.*—An impression on the afferent nerves of a given part will cause a variation in the arteries of all organs situated directly beneath that part.

*Third.*—In the case of organs which are in pairs and perfectly symmetrical, as the eyes, ears, hands, and feet (the lungs, kidneys, and testicles are not,) variations in the calibre of the arteries of one will cause a similar variation in the other.

*Fourth.*—Variations in the calibre of the arteries of certain parts are accompanied by corresponding changes in the arteries of certain other parts, and these particular associations are to be determined by experiment: for example, the relation between the circulation of the feet and that of the pelvic viscera and the pharynx, and the relation of the circulation at the nape of the neck to that of the head and face.

The following instances will suffice to illustrate the application of these laws in the use of cold:

1. Cold water applied directly to a bleeding surface.
2. Ice-bags to the epigastrium to check hæmatemesis.
3. Holding any cold body in one hand to arrest hemorrhage in the other.
4. Cold foot-baths to arrest metrorrhagia.

In post-partum hemorrhage the best means of applying cold is by ether spray, for the sudden and intense impression produced causes effectual contraction of the uterus without chilling the patient. If ether spray is not available, cold water should be poured upon the abdomen from a height of two or three feet, the shock of the falling water materially assisting the action of the cold. Either of the above measures may be used for hæmoptysis.

COLD AS AN ANTIPHLOGISTIC.

As an antiphlogistic, cold may be used to arrest an acute inflammation, unless suppuration has occurred, or to prevent inflammation when threatened. This it does by causing a protracted constriction of the arteries, thereby preventing the active congestion essential to all acute inflammation. It should be invariably applied as dry cold, directly to the part affected, in sufficient intensity to relieve pain, and continued so long as the exciting cause exists. If, before the tendency to inflammation has entirely disappeared, a neuralgic pain occurs, it is a sign that the vaso-motor nerves have become exhausted, and the use of cold must at once be discontinued, or gangrene will result; moreover, the patient will feel more comfortable without than with the cold applications. This neuralgic pain is continuous, and, if the injured part be one of the extremities, it extends from the part injured toward the trunk. Inflammatory pain, on the other hand, is local throbbing, accompanied by local heat, and is relieved by more thorough applications of cold. In fractures, or other severe injuries near joints, the injured parts should be surrounded with pounded ice placed in pigs' bladders or rubber bags, two or three layers of perfectly dry muslin being placed between the skin and bags, lest the parts be chilled too suddenly. A bottle filled with ice-water makes a good antiphlogistic splint for injuries of the hand. Inflammation of the eyes may be controlled, and its spread from one eye to the other prevented, by means of cold applications. Ice-bags should be applied to the head and spine in epidemic cerebro-spinal meningitis. Cold applications will control the spread of erysipelas, and are the best means for relieving febrile headache. Headache from uterine trouble is best relieved by moist warmth. Cold should not be used antiphlogistically in any acute inflammation of internal organs except peritonitis with vomiting, and meningitis.

### COLD AS AN ANÆSTHETIC.

The use of cold as an anæsthetic depends upon its physical property of freezing tissue and deadening sensation without injuring vitality. It is most useful in operations where no great thickness of tissue is involved, as in opening abscesses, amputation of fingers, Cæsarean section, and ovariotomy. In all cases the action of the cold should be secured as rapidly as possible. Apply ether spray to the part alone which is to be operated upon. Anæsthesia is complete as soon as the skin becomes white and glistening.

### COLD AS AN ANTIPYRETIC.

When the abnormal elevation of the bodily temperature is due to insufficient radiation of heat, as in some nervous disorders, it is not generally in itself dangerous; for it has been known to reach 123° F., and remain there for several weeks. But if, as in fevers, the rise of temperature depends upon excessive chemical changes, then the heat itself is injurious, causing arrest of gland-secretion, as well as extensive destruction of tissue. In every fever there is a certain point beyond which, if the temperature rises, certain structural changes will take place. The glands become affected with cloudy swelling, and fatty degeneration ensues, and the muscles affected in the same manner become remarkably brittle.

The point at which these changes occur differs in each fever. In scarlet fever it is 105° F.; in typhoid fever 106° F.; in relapsing fever from 107° to 108° F.; and in erysipelas still higher. Beyond this dangerous point in each fever the temperature should not be allowed to rise, but must be lowered by the use of cold, the result of which is simply the abstraction of heat. This may be effected by immersion in a cold bath or by the cold pack. Place the patient in a bath at 75° F., and gradually cool the water down to 65° or 60° F.—never lower, and at the same time use cold effusions to the head continuously. At first the temperature will rise slightly, owing to the blood being driven from the surface of the body into the viscera, which are always a little warmer than the skin; but the bath should be continued until the temperature is reduced to 100° F., provided the fall is gradual—that is, one degree in six, five, four, or three minutes. If it falls one degree in two and a half minutes, stop the bath when the temperature has reached 101° F.; for in most cases a further reduction of one degree will occur after the bath is discontinued. If the fall in temperature during the bath be one degree in *two* minutes, the patient should be taken out at once, whatever the actual temperature may be; for in such cases there is danger of the subsequent fall becoming uncontrollable, reaching perhaps 97° F., and the patient passing into collapse. Should this at any time occur, wrap the patient in hot blankets, apply hot saucers to the epigastrium, and give brandy or other stimulants.

When, for any reason the bath is impracticable, the cold pack may be used, always, however, with the same precautions as in the use of the cold bath. First wrap the patient in a sheet wrung out of water at an ordinary temperature, say 70° F., and then lay on other sheets wrung out of ice-water. The cold bath or pack should be repeated often enough to keep the temperature below the point of danger for that particular disease. If necessary, use one every hour. If, however, two or three a day are sufficient, one should be so timed as to be given just before the highest rise of the fever-heat—that is, usually between two and three o'clock in the afternoon.

The contra-indications to the antipyretic use of cold are hemorrhage from the bowels and notable variations of temperature from the regular course. Bronchitis and pneumonia are not necessarily contra-indications.—*Med. Record.*

